# NOD2‐mediated HDAC6/NF‐κb signalling pathway regulates ferroptosis induced by extracellular histone H3 in acute liver failure

**DOI:** 10.1111/jcmm.17582

**Published:** 2022-10-12

**Authors:** Qian Chen, Qingqi Zhang, Pan Cao, Chunxia Shi, Luyi Zhang, Luwen Wang, Zuojiong Gong

**Affiliations:** ^1^ Department of Infectious Diseases Renmin Hospital of Wuhan University Wuhan China

**Keywords:** ALF, HDAC6, histone H3, NF‐κb, NOD2

## Abstract

Acute liver failure (ALF) is life‐threatening and often associated with high mortality rates. The aim of the present study was to investigate whether extracellular histone H3 could induce ferroptosis in hepatic macrophages in ALF and explore its potential mechanism. RAW264.7 macrophages and C57BL/6 mice were used in this study. LPS, D‐galactosamine (D‐Gal), histone H3, histone H3 antibody, NOD2 agonist Muramyl Dipeptide (MDP) and HDAC6‐siRNA were administered in this study. The key molecules of ferroptosis, NOD2, HDAC6 and the NF‐κb pathway, were detected. In vitro, histone H3 was released into the extracellular environment from cell nucleus after LPS exposure. In addition, histone H3 could induce ferroptosis in RAW264.7 macrophages with increased level of Fe^2+^ and ROS and decreased levels of GPX4 and GSH. MDP further aggravated ferroptosis in RAW264.7 macrophages stimulated by histone H3, which was accompanied by elevated NOD2, HDAC6, p‐P65 and IκBα. HDAC6‐siRNA ameliorated ferroptosis in RAW264.7 macrophages induced by histone H3, which was accompanied by decreased levels of HDAC6, p‐P65 and IκBα. However, HDAC6‐siRNA did not alter NOD2 levels in RAW264.7 macrophages administered histone H3. In vivo, the levels of NOD2, HDAC6 the NF‐κb pathway and ferroptosis were increased in ALF mice, which were downregulated by histone H3 antibody and upregulated by histone H3. Extracellular histone H3 could induce ferroptosis in hepatic macrophages in ALF by regulating theNOD2‐mediated HDAC6/NF‐κb signalling pathway.

## 1 INTRODUCTION

Acute liver failure (ALF) is a severe emergent hepatic condition and usually has a poor prognosis.^1^ Massive hepatic necrosis, variable inflammation and bile duct proliferation are typical pathological features of ALF.^2^ Studies have shown that extracellular histones are implicated in the pathogenesis of various diseases, such as sepsis and acute liver and kidney injury.^3^ Histone release in sepsis affects the types of cells that can even contribute to fatal organ dysfunction.^4^ Specifically, histone H3 exhibits cytotoxic activity by acting on phospholipid bilayers of cellular membranes which alters channel formation and leads to the loss of membrane barrier function.^5^ Moreover, recent studies have revealed that histoneH3 can activate the Toll‐like receptor signalling pathway, leading to NF‐κB activation and downstream cytokine production, an inflammatory response and tissue damage.^6^

Then, nucleotide‐binding and oligomerization domain 2 (NOD2) is known as an intracellular bacterial sensing receptor.^7^ In addition, the activation of NOD2 triggered by MDP can drive multitudinous inflammatory responses that are dependent on the activation of the NF‐κB and MAPK signalling pathways.^8^ Histone deacetylases 6 (HDAC6) is implicated in cell survival, cell cycle progression and many biological processes.^9^ In addition, HDAC6 inhibition blocks NF‐κB signalling to alleviate LPS‐induced inflammation in macrophages.^10^

Ferroptosis is an iron‐dependent cell death manner mechanism that is manifested by the accumulation of iron and reactive oxygen species in cells, resulting in excessive oxidative stress and membrane lipid peroxidation.^11^ Macrophages are a group of immune cells that can mediate inflammation and iron and lipid metabolism. In addition, the regulation of Kupffer cells can influence the development of ferroptosis, providing a new possible therapy for NAFLD.^12^ In addition, ferroptosis and the NF‐κB signalling pathway are closely related to the development of many inflammatory diseases.^13^ Chang et al first proved that an IκBα inhibitor suppressed viability in cancer cells by regulating ferroptotic death in an NF‐κB‐independent manner.^14^ In recent years, it has been found that ferroptosis plays a critical regulatory role in liver diseases.^15^ Yamada et al demonstrated that ferroptosis triggered by ω‐6 PUFAs is associated with acetaminophen‐induced acute liver injury.^16^ In addition, chronic HCV infection favours the accumulation of iron in liver tissue, and progressive iron overload further worsens liver damage.^17^ Our previous study found that the ferroptosis pathway is activated in ALF mice.^18^ At present, there is no report on the influence of extracellular histoneH3 on ferroptosis in ALF. Moreover, the NF‐κB signalling pathway is a crucial link in many inflammatory diseases and is tightly related to the ferroptosis. It was proven that NOD2 and HDAC6 can regulate the NF‐κB signalling pathway. This study aimed to investigate whether the extracellular histone H3 could regulate ferroptosis in the process of ALF and explore the relationship among the NOD2, HDAC6 and NF‐κB signalling pathways.

## 2 MATERIALS AND METHODS

### 2.1 Reagents and antibodies

D‐Gal, LPS and calf thymus histone H3 were purchased from Sigma. HDAC6‐siRNA was obtained from RiboBio. The NOD2 agonist MDP and Lipofectamine 2000 Transfection Reagent were purchased from InvivoGen. Antibodies againstH3, NOD2, HDAC6, P65, p‐P65, IκBα and GPX4 was obtained from Cell Signaling Technology. GAPDH antibody were purchased from Proteintech. The PrimeScriptTM RT reagent kit and SYBR Premix Ex Taq kit were purchased from TaKaRa (Japan). Dulbecco's modified Eagle's medium (DMEM) and foetal bovine serum (FBS) were purchased from Gibco (NY, USA). The H3 ELISA Kit was obtained from Bioswamp. The glutathione (GSH) assay kit was obtained from Naijing Jiancheng Bioengineering Institute. Reactive oxygen species (ROS) kits were obtained from Beyotime Institute of Biotechnology. An iron assay kit was purchased from Abcam.

### 2.2 Establishment of mouse models

In total, 40 male specific pathogen‐free (SPF) C57BL/6 mice (20–25 g) were purchased from the Experimental Animal Center of Wuhan University. The protocols of this study were approved and consented to by the Ethics Committee of Renmin Hospital of Wuhan University. The mice were nourished in the animal experiment centre in Renmin Hospital of Wuhan University with a humidity of 50% ± 15% at 20–25°C and a 12 h light/dark cycle. After 7 days, 40 mice were divided randomly into 4 groups: the normal (*n* = 10), model (*n* = 10), H3 (*n* = 10) and H3 antibody groups (*n* = 10). Mice in the model group, H3 group and H3 antibody group were induced by an intraperitoneal injection of D‐Gal (400 mg/kg) and LPS (100 μg/kg). The normal group was given the same injection amount but of normal saline. Histone H3 (25 mg/kg)^19^ and H3 antibody (10 mg/kg)^19^ were given by intraperitoneal injection to H3 group and H3 antibody group, respectively, 2 h before the ALF model protocol. All experimental animals were sacrificed under anaesthesia after 24 h when the ALF model was established.

### 2.3 Cell culture and transfection

RAW264.7 cells were cultured in DMEM supplemented with 10% FBS at 37 °C in an incubator containing 5% CO_2._ First, the cells were divided into normal and LPS groups to explore whether LPS could induce the release of intranuclear histone H3 into the extracellular environment. Next, the cells were divided into a normal group and an H3 group to investigate whether extracellular histone H3 could induce ferroptosis in RAW264.7 cells. Then, the cells were divided into normal, LPS and H3 antibodies to detect the levels of ferroptosis, NOD2, HDAC6 and key molecules of the NF‐κB signalling pathway. Finally, the cells were divided into normal, H3, MDP, HDAC6‐siRNA and MDP + HDAC6‐siRNA groups to investigate the potential regulatory relationship among the NOD2, HDAC6 and NF‐κB signalling pathways and ferroptosis. LPS (1 μg/ml) and histone H3 (50 μg/ml)^4^ were added to the medium for 24 h in LPS group and H3 group. Histone H3 antibody (10 μg/ml)^20^ was applied to the medium 2 h before LPS (1 μg/ml) was added to the in H3 antibody group. HDAC6‐siRNA transfection was performed24 h prior to H3 stimulation and MDP (1 μg/ml)^21^ was added to the medium 2 h prior to H3 stimulation in the HDAC6‐siRNA, MDP and MDP + HDAC6‐siRNA groups. The transfection was accomplished using Lipofectamine 2000 (Invitrogen) according to the manufacturer's instructions.

### 2.4 Histopathological analysis and biochemical examination

Fresh liver specimens were fixed using 10% neutral‐buffered formalin for 1 day. Next, they were embedded in paraffin, processed for sectioning and stained with haematoxylin–eosin (H&E).^22^ Liver sections were observed under a BX 51 light microscope (Olympus). The serum ALT and AST levels in each group were detected by an automated Aeroset chemistry analyser (Abbott Co. Ltd.).

### 2.5 Immunofluorescence detection of H3 and GPX4 protein expression

A total of 1 × 10^4^ L02 cells/500 μl per plate were seeded in 24‐well plates, and circular slides were placed. After treatments, the slides were fixed with 4% paraformaldehyde at 37°C for 30 min, permeabilized with 0.2% Triton X‐100 at 37°C for 15 min, blocked with 5% BSA at 37°C for 1 h and incubated with primary antibodies against H3 (1:100) and GPX4 (1:100) at 4°C overnight. Slides were then incubated with Cy3‐labelled fluorescent secondary antibodies at 37°C for 1 h in dark room. Then, the slides were stained with 5 μg/ml DAPI at 37°C for 3 min in a dark environment. The slides were observed under a confocal microscope (Olympus, #FV1200) at 1000× magnification.

### 2.6 Western blotting

The liver tissues or RAW264.7 cells were homogenized in radioimmunoprecipitation assay (RIPA) lysis buffer on ice to extract total protein. The protein concentration was detected by bicinchoninic acid (BCA) protein assay reagent assay kit (Beyotime Institute of Biotechnology). Protein lysates (50 μg) were subjected to 12% SDS–PAGE. The proteins were transferred to PVDF membranes. After blocking with 20% nonfat milk for 1 h, the membranes were incubated with primary antibodies against NOD2 (1:1000), HDAC6 (1:1000), P65 (1:1000), p‐P65 (1:1000), IκBα (1:1000), GPX4 (1:1000) and GAPDH (1:2000) overnight at 4°C. All the above bands were incubated with the IRDye 800CW secondary antibody (1:15,000) for 1 h. The bands were observed by the Odyssey Infrared Imaging System.

### 2.7 Quantitative real‐time PCR

Total RNA in RAW264.7 cells and mouse liver tissues were isolated using RNAiso Plus. Then, the RNA samples were reverse transcribed into cDNA using a Prime‐Script RT reagent kit. qRT–PCR was performed using a SYBR Premix Ex Taq kit on a StepOnePlus device. All data were measured by the 2^−ΔΔCT^ method. All the primers were synthesized by Tsingke, and the sequences are shown in Table 1.

**TABLE 1:** The primers utilized for amplification of the respective genes

Gene		Primer sequences (5′–3′)
NOD2	Forward	CCTAGCACTGATGCTGGAGAAG
	Reverse	CGGTAGGTGATGCCATTGTTGG
HDAC6	Forward	TCGCTGTCTCATCCTACCTGCT
	Reverse	GTCAAAGTTGGCACCTTCACGG
GPX4	Forward	CCTCTGCTGCAAGAGCCTCCC
	Reverse	CTTATCCAGGCAGACCATGTGC
GAPDH	Forward	CATCACTGCCACCCAGAAGACTG
	Reverse	ATGCCAGTGAGCTTCCCGTTCAG

### 2.8 Detection of ROS

According to the instructions, DCFH‐DA was diluted with serum‐free culture medium at a ratio of 1:1000 to a final concentration of 10 μmol/L. Then, the cell culture medium was removed and add 1 ml of DCFH‐DA was added to each well of the six‐well plate. The cells were incubated in a 37 °C cell incubator for 20 minutes. Then, the cells were washed three times with serum‐free cell culture medium to fully remove the DCFH‐DA that had not entered the cells. Finally, animal cells are detected with a fluorescence microplate reader. RAW264.7 cells were measured with a flow cytometer.

### 2.9 Detection of H3, GSH and Fe^2+^

The H3, GSH and Fe^2+^ kits were used according to the corresponding manufacturer's instructions. The levels of H3, GSH and Fe^2+^ in RAW264.7 cells and liver tissue were detected with a multimode plate reader.

### 2.10 Statistical analysis

Measurement data are expressed as the mean ± SD. The experimental data were compared by one‐way analysis of variance (anova). When *p* < 0.05, differences were considered statistically significant. Statistical analysis was performed using GraphPad Prism 8.0.

## 3 RESULTS

### 3.1 LPS induced the release of histone H3 and H3 stimulated ferroptosis in RAW264.7 cells

**FIGURE 1:**
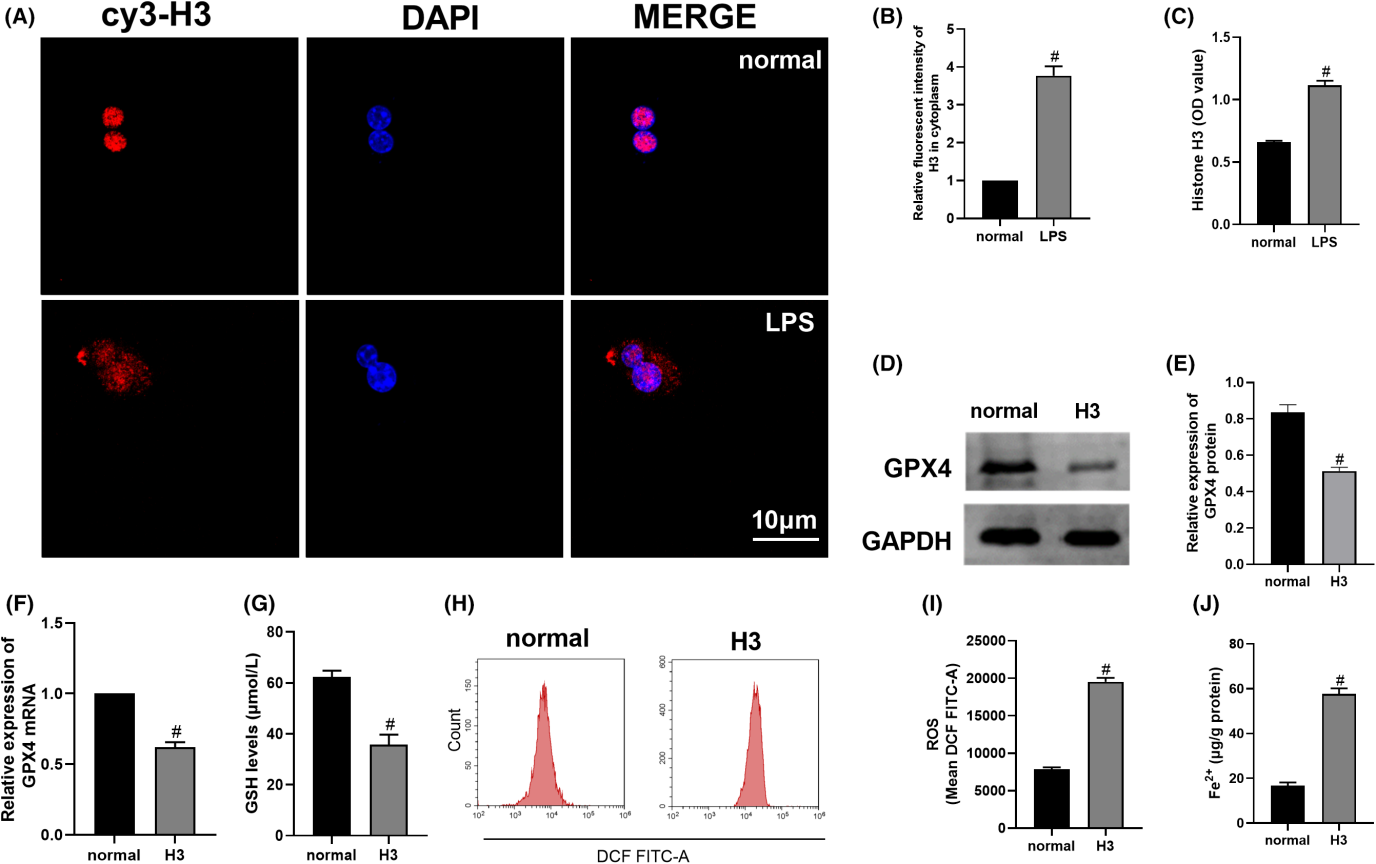
Histone H3 was released in RAW264.7 cells stimulated by LPS and extracellular histone H3 induced ferroptosis in RAW264.7 cells. (A, B) The expression of H3 in RAW264.7 cells observed by confocal microscopy. (C) H3 level in the cell supernatant detected by ELISA. (D‐E) Western blotting for GPX4 in RAW264.7 cells. (F) The mRNA level of GPX4 in RAW264.7 cells measured by Quantitative real‐time PCR. (G–J) GSH, ROS and Fe^2+^ levels in RAW264.7 cells detected by kits. The cell experiment of Western blotting was repeated 3 times and other experiments were repeated 6 times. Compared with the normal group, ^#^ *p* < 0.05.

First, LPS was used to stimulate RAW264.7 cells to explore whether histone H3 was released into the extracellular environment. As shown in Figure 1A–C, compared with the normal group, cytoplasmic and extracellular histone H3 levels were significantly increased in the LPS group (*p* < 0.05). The above results proved that histone H3 is released into the extracellular environment when RAW264.7 cells are induced by LPS. As shown in Figure 1D–G, the GPX4 mRNA, protein and GSH levels were reduced in the H3 group compared with the normal group (*p* < 0.05). As shown in Figure 1H–J, compared with the normal group, the ROS and Fe^2+^ levels were significantly increased in the H3 group (*p* < 0.05). The data showed that extracellular H3 triggered ferroptosis as well.

### 3.2 Histone H3 antibody inhibited ferroptosis in LPS‐stimulated RAW264.7 cells

**FIGURE 2:**
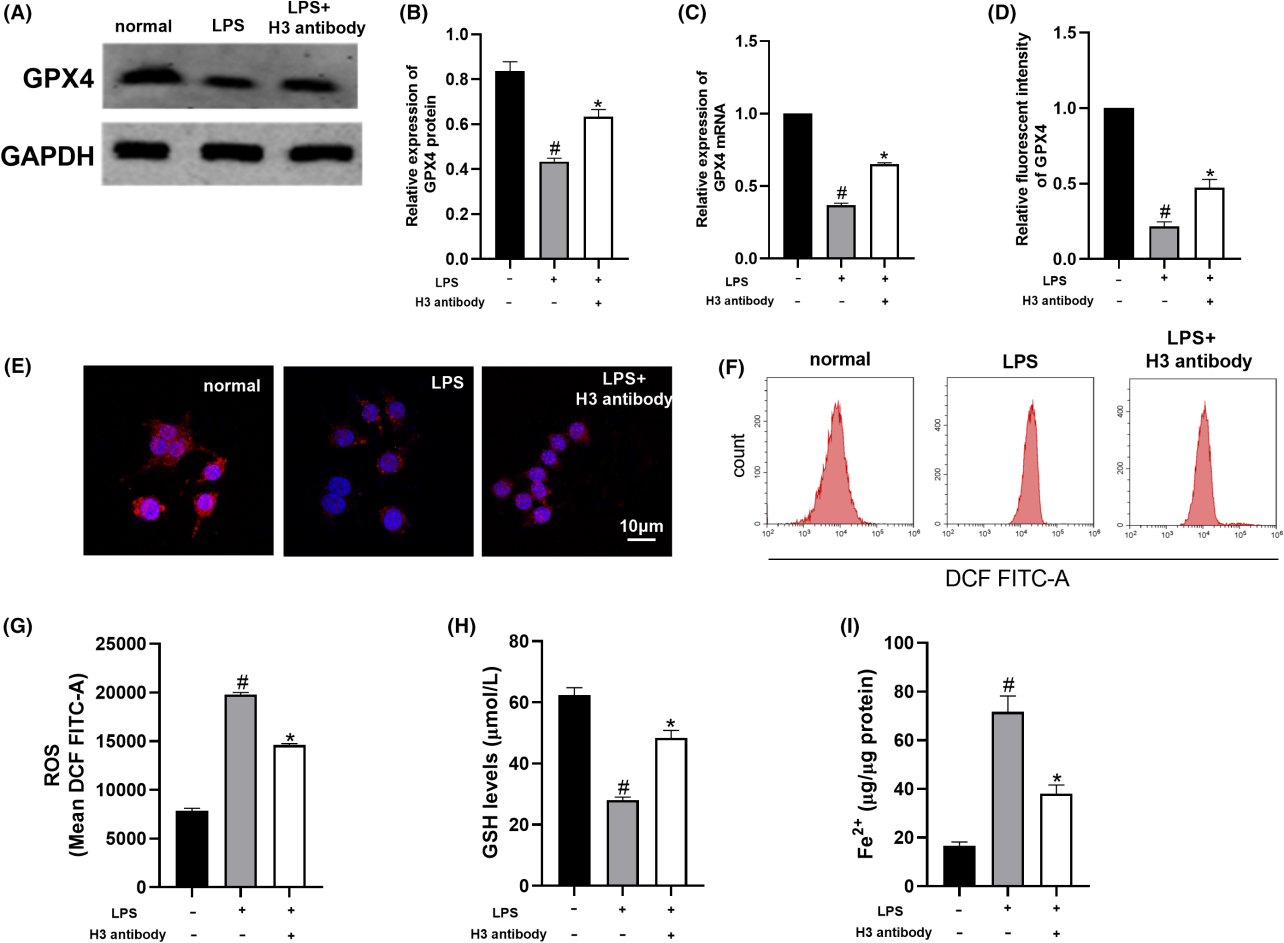
Histone H3 antibody reduced the level of ferroptosis in RAW264.7 cells induced by LPS. (A, B) GPX4 protein level detected by Western blotting. (C) Quantitative real‐time PCR for GPX4 mRNA levels. (D, E) Immunofluorescence analysis of GPX4 levels. (F–I) ROS, GSH and Fe^2+^ levels in RAW264.7 cells detected by kits. The cell experiment of Western blotting was repeated 3 times and the other experiments were repeated 6 times. Compared with the normal group, ^#^ *p* < 0.05; compared with the LPS group, *p < 0.05.

As shown in Figure 2A–E,H, LPS suppressed the expression of GPX4 and GSH in RAW264.7 cells (*p* < 0.05). In addition, ROS and Fe^2+^ levels were significantly increased in RAW264.7 cells induced by LPS (*p* < 0.05) (Figure 2F,G,I). Compared with the LPS group, GPX4 and GSH levels were elevated after treatment with histone H3 antibody (*p* < 0.05). Moreover, ROS and Fe^2+^ levels in the LPS group were markedly reduced after treatment with histone H3 antibody (*p* < 0.05). These results indicated that ferroptosis was induced by LPS in RAW264.7 cells. The histone H3 antibody suppressed ferroptosis in RAW264.7 cells stimulated with LPS.

### 3.3 The effect of histone H3 antibody on the NOD2, HDAC6 and NF‐κB signalling pathways in LPS‐stimulated RAW264.7 cells

**FIGURE 3:**
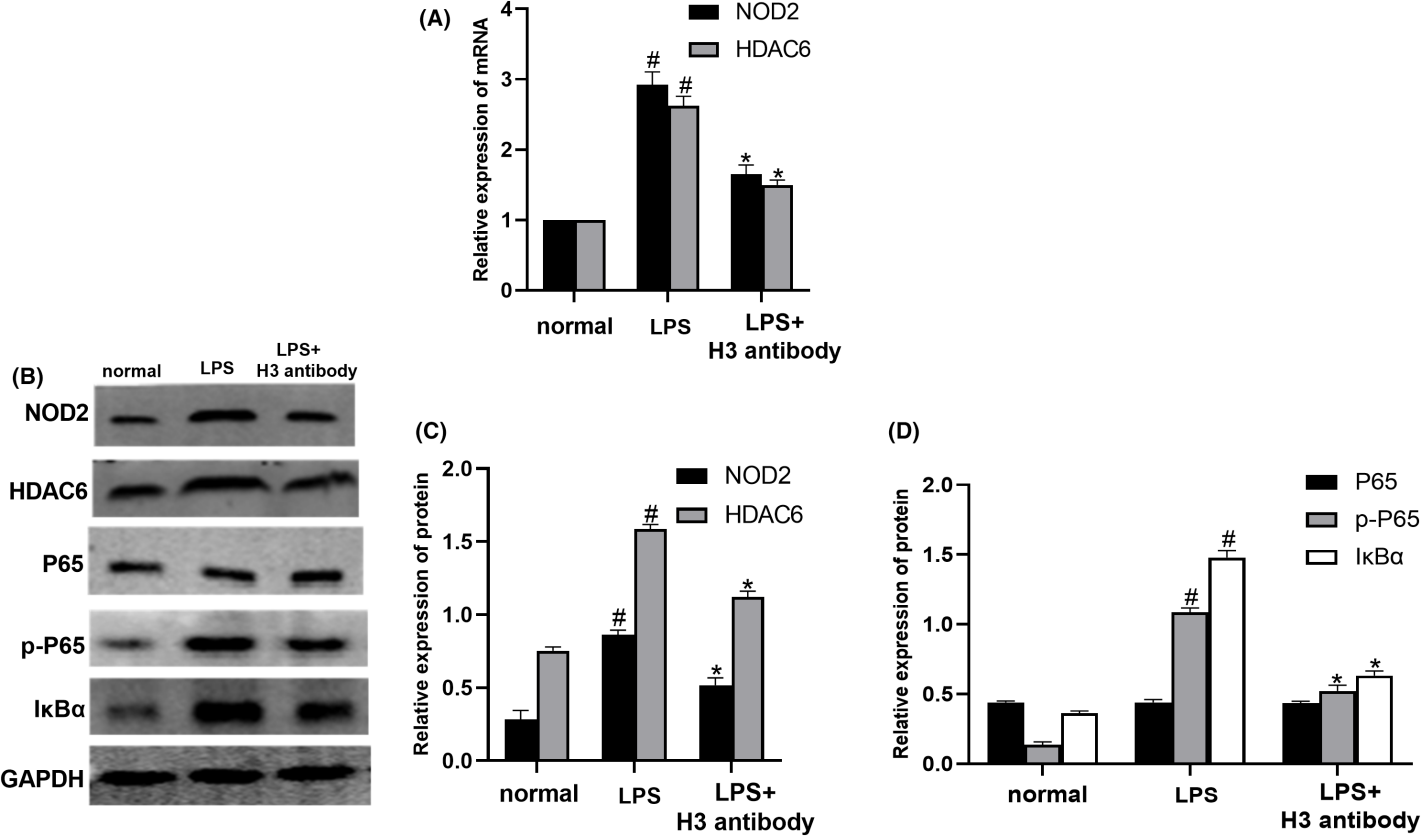
Histone H3 antibody inhibited the expression of NOD2, HDAC6, p‐P65 and IκBα in LPS‐induced RAW264.7 cells. (A) NOD2 and HDAC6 mRNA levels measured by quantitative real‐time PCR. (B–D) NOD2, HDAC6, P65, p‐P65 and IκBα protein levels in RAW264.7 cells detected by Western blotting. The cell experiment of Western blotting was repeated 3 times and quantitative real‐time PCR was repeated 6 times. Compared with the normal group, ^#^ *p* < 0.05; compared with the LPS group, *p < 0.05.

As shown in Figure 3A–D, compared with the normal group, NOD2 and HDAC6 mRNA levels and NOD2, HDAC6, p‐P65 and IκBα protein levels were significantly increased in the LPS group (*p* < 0.05). After treatment with histone H3 antibody, NOD2 and HDAC6 mRNA levels and NOD2, HDAC6, p‐P65 and IκBα protein levels were markedly decreased in the LPS group (*p* < 0.05). These data indicated that the levels of the NOD2, HDAC6 and NF‐κB signalling pathways were elevated in LPS‐stimulated RAW264.7 cells. However, the histone H3 antibody inhibited those levels in RAW264.7 cells induced by LPS.

### 3.4 The relationship between NOD2 and HDAC6 in RAW264.7 cells

**FIGURE 4:**
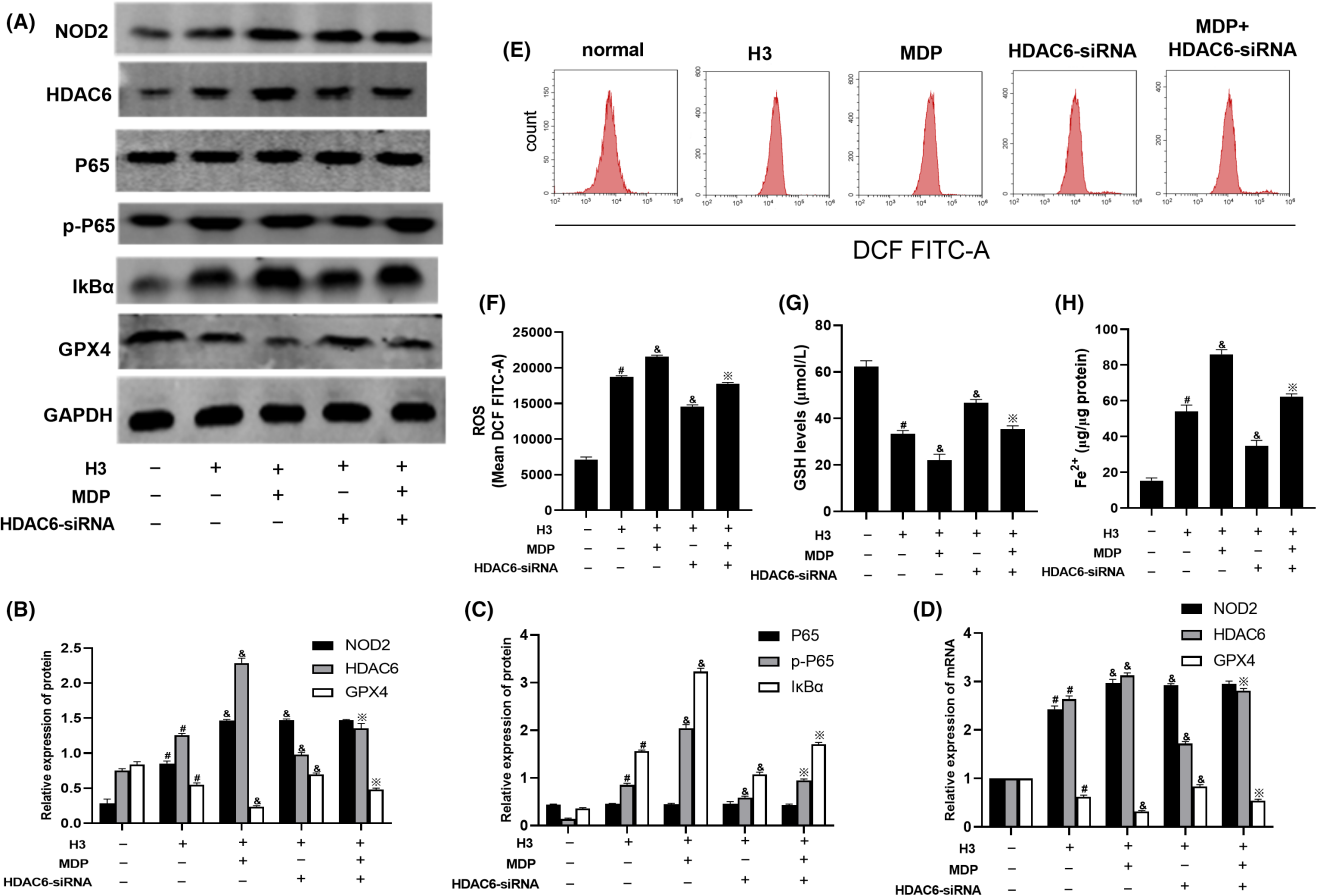
The relationship between NOD2 and HDAC6 and the effect of MDP and HDAC6‐siRNA on NF‐κB signalling pathway and ferroptosis. (A–C) NOD2, HDAC6, P65, p‐P65 and IκBα protein levels in RAW264.7 cells were determined by Western blotting. (D) Quantitative real‐time PCR for NOD2 and HDAC6mRNA levels. (E–H) ROS, GSH, and Fe^2+^ levels in RAW264.7 cells detected by kits. The Western blotting experiment was repeated 3 times and the other experiments were repeated 6 times. Compared with the normal group, ^#^ *p* < 0.05; compared with the H3 group, ^&^ *p* < 0.05. compared with the MDP group, ^※^ *p* < 0.05.

To investigate whether there was a connection between NOD2 and HDAC6, the NOD2 agonist MDP and HDAC6‐siRNA were used. As shown in Figure 4A,B,D, NOD2 and HDAC6 mRNA and protein levels were increased in the H3 group compared with the normal group (*p* < 0.05). MDP further upregulated NOD2 and HDAC6 levels in the H3 group (*p* < 0.05). HDAC6‐siRNA decreased t HDAC6 levels in the H3 group (*p* < 0.05). However, there was no significant difference in NOD2 levels between the H3 group and the and HDAC6‐siRNA group. of the NOD2 mRNA and protein levels in the MDP group and the MDP + HDAC6‐siRNA group were not significantly different. However, t HDAC6 mRNA and protein levels in the MDP group were increased compared with those in the MDP + HDAC6‐siRNA group (*p* < 0.05). These results indicated that NOD2 may act on the upstream signalling molecule that could regulate the expression of HDAC6. The NF‐κB signalling pathway and ferroptosis were also assessed. As shown in Figure 4C,E–H, MDP also upregulated p‐P65, IκBα, Fe^2+^ and ROS levels in the H3 and HDAC6‐siRNA groups (*p* < 0.05). The expression of GPX4 and GSH was decreased in the MDP group compared with the H3 and HDAC6‐siRNA + MDP groups. In addition, HDAC6‐siRNA downregulated p‐P65, IκBα, Fe^2+^ and ROS levels and increased GPX4 and GSH levels in the H3 group (*p* < 0.05). These results verified the effect of NOD2 and HDAC6 on the NF‐κb signalling pathway and ferroptosis.

### 3.5 Histone H3 antibody attenuated and histone H3 deteriorated liver pathological changes and liver function in ALF mice

**FIGURE 5:**
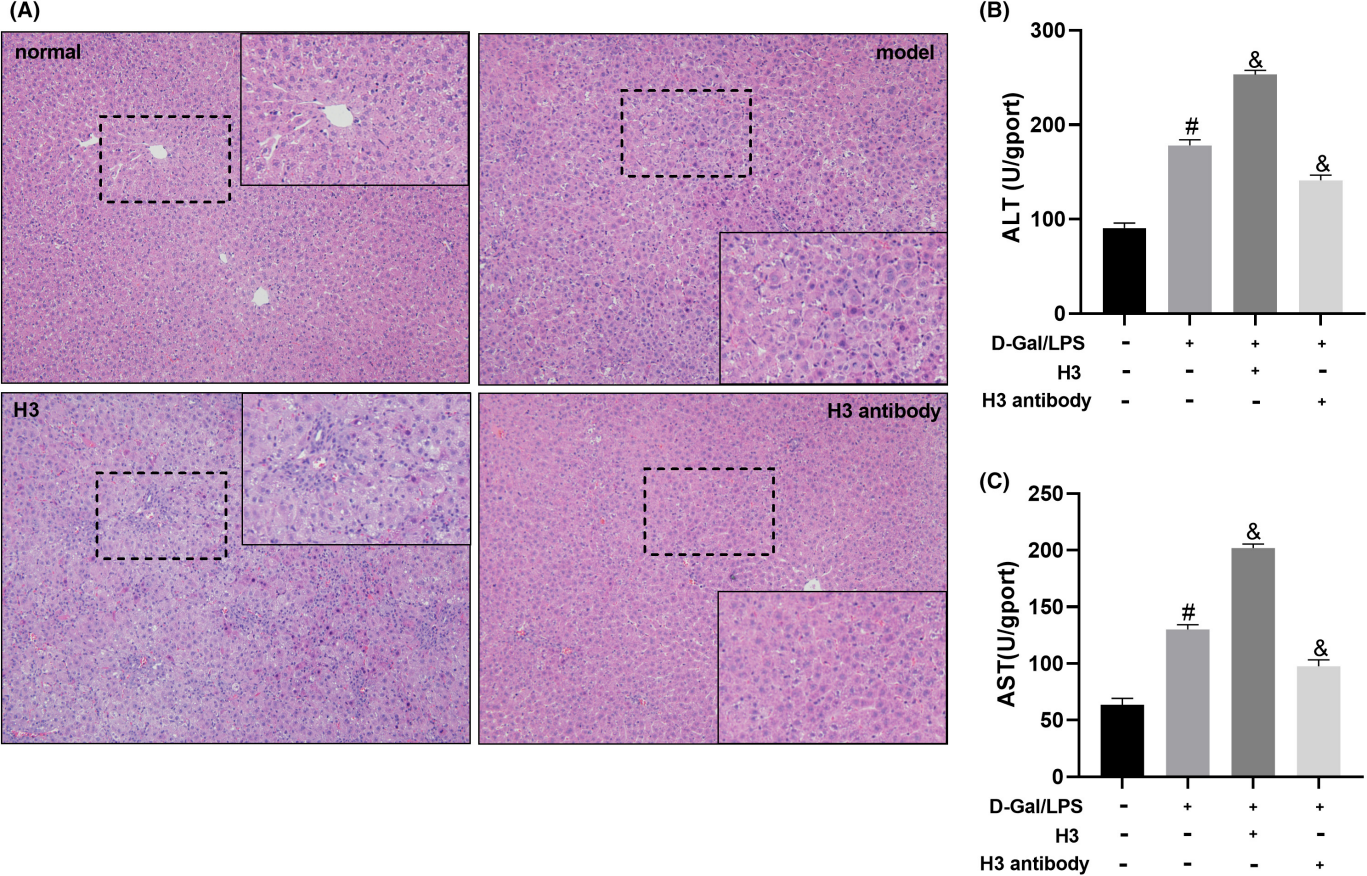
The effect of histone H3 and H3 antibodies on pathological changes in liver tissue and liver function in mice. (A) Pathological changes in the mouse liver tissue were observed by H&E staining. (B, C) ALT and AST serum levels in mice. The mouse experiment was repeated 6 times. Compared with the normal group, ^#^ *p* < 0.05; compared with the model group, ^&^ *p* < 0.05.

As shown in Figure 5A, compared with the normal group, the hepatocytes were necrotic and the structure of liver tissue was disordered in D‐Gal/LPS‐induced ALF mice. Moreover, compared with the model group, hepatic necrosis was more evident in the liver tissue, which was accompanied by a mass of inflammatory cell infiltration in the H3 group. However, after histone H3 antibody treatment, the degree of hepatocytes was reduced compared with that in the model group. As shown in Figure 5B,C, ALT and AST serum levels in the model group were remarkably higher than those in the normal group (*p* < 0.05). Additionally, histone H3 further increased and histone H3 antibody reduced level of ALT and AST serum levels in the model group (P < 0.05).

### 3.6 Histone H3 aggravated and Histone H3 antibody inhibited ferroptosis in ALF mice through theNOD2‐mediated HDAC6/NF‐κb signalling pathway

**FIGURE 6:**
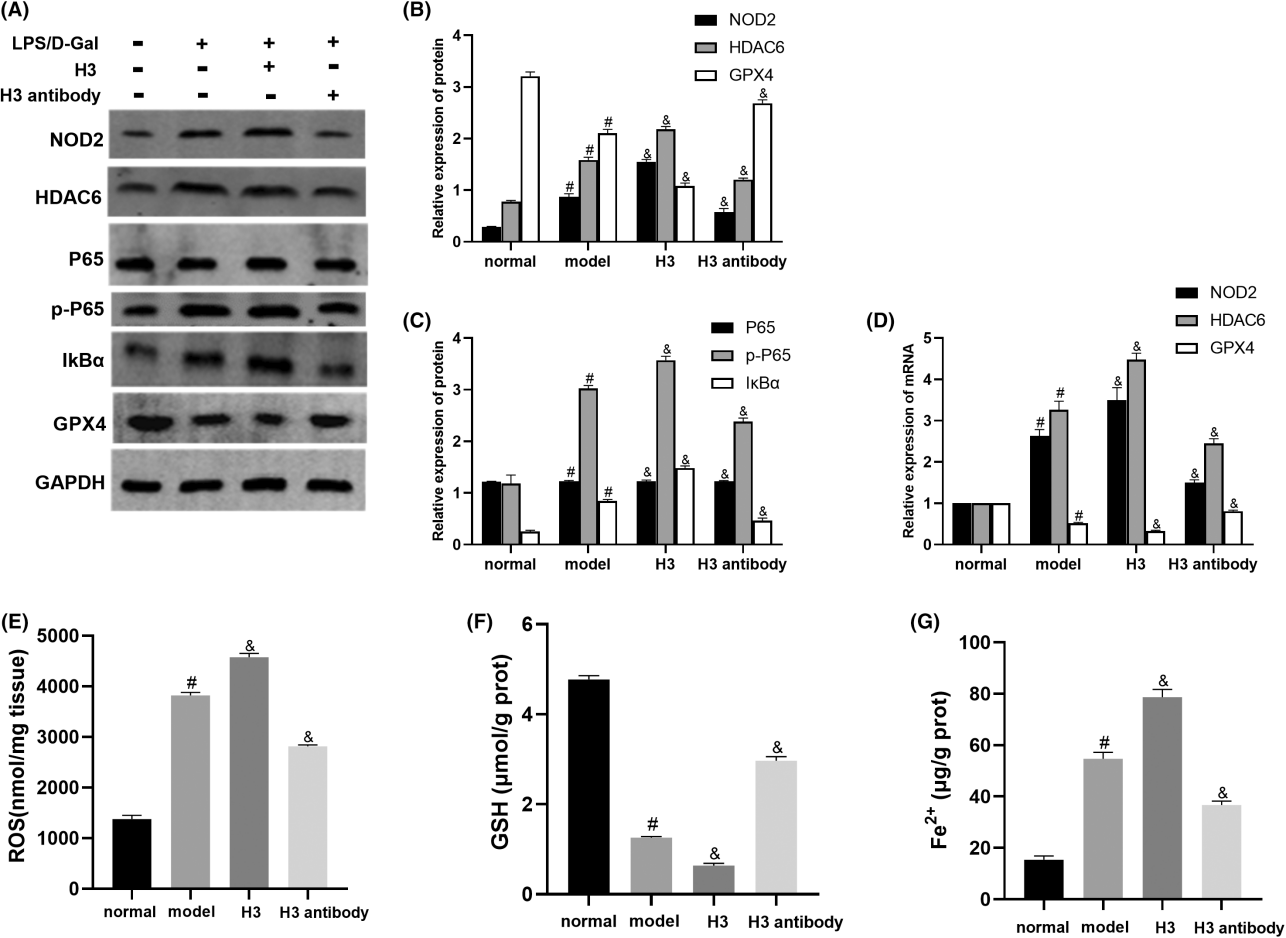
The effect of histone H3 and H3 antibodies on the expression of the NOD2, HDAC6 and NF‐κB signalling pathways and ferroptosis in ALF mice. (A–C) Western blotting for NOD2, HDAC6, P65, p‐P65, IκBα and GPX4 protein levels in mice. (D) Quantitative real‐time PCR for NOD2, HDAC6 and GPX4 mRNA levels. (E–G) ROS, GSH and Fe^2+^ levels in mice detected by kits. The mouse experiment of Western blotting was repeated 3 times and the other experiments were repeated 6 times. Compared with the normal group, ^#^ *p* < 0.05; compared with the model group, ^&^ *p* < 0.05.

As shown in Figure 6A,B, NOD2 and HDAC6 mRNA and protein levels in the model group were significantly increased compared with those in the normal group, which were reduced by the histone H3 antibody and further increased by histone H3 (*p* < 0.05). As shown in Figure 6A–E and Figure 6G, the expression of p‐P65, IκBα, Fe^2+^ and ROS showed results similar to those of NOD2 and HDAC6 (*p* < 0.05). However, GPX4 and GSH levels were lower in the model group than in the normal group (*p* < 0.05). Histone H3 antibody upregulated and histone H3 downregulated GPX4 and GSH level in the model group (*p* < 0.05) (Figure 6A,B,D,F).

## 4 DISCUSSION

ALF is a rare and severe disease that can rapidly become fatal.^23^ Severe liver damage in ALF is caused by various factors. Viral infection and drug toxicity are common pathogenic factors. Medication, artificial liver replacement therapy and liver transplantation surgery are the dominant treatments for ALF.^24^ At present, liver transplantation is the most effective treatment. However, its application is restricted because of the high associated costs and a shortage of donor organs. Ferroptosis is a type of cell death characterized by iron homeostasis and ROS prodution.^25^ In the key process of ferroptosis, Fe^2+^ converts lipid peroxide into ROS. In addition, GPX4 converts lipid peroxide into corresponding alcohol with the assistance of GSH. Moreover, studies have shown that the changes in the of ROS, Fe^2+^, GSH and GPX4 levels can be combined to define ferroptosis.^18^ Accumulating studies have proven that ferroptosis has a vital role in the pathogenesis of various diseases such as cardiovascular disease and kidney and liver diseases.^26^, ^27^, ^28^ Our data showed that Fe^2+^ and ROS levels were increased in ALF. The expression of GPX4 and GSH was decreased. These results indicated that ferroptosis is triggered in ALF. Once the level of ferroptosis was reduced, liver injury was alleviated.

Histones are crucial structural members of nuclear chromatin and control gene transcription.^29^ However, ALF patients and animals have high levels of circulating histones.^30^ To better study the effect of extracellular histone H3 in ALF, we established an animal model of D‐Gal/LPS‐induced hepatic injury in vivo. We found that histone H3 could aggravate the damage to liver structure and function, but histone H3 antibody improved liver function. In addition, Xu et al also demonstrated that toxic liver injury causes histone release from hepatocytes, which can be prevented with anti‐histone antibodies.^19^ Moreover, extracellular histones can lead to multiple organ injuries by killing endothelial cells and eliciting immunostimulatory effects.

In the current study, the results showed that histone H3 was actively released in RAW264.7 cells stimulated by LPS. LPS activated the ferroptosis pathway which presented as increased levels of Fe^2+^ and ROS and decreased levels of GSH and GPX4. Histone H3 antibody inhibited the increased ferroptosis level in LPS‐treated RAW264.7 cells and ALF model mice. The NOD2, HDAC6, p‐P65 and IκBα levels were increased in the cell model and animal groups compared with the normal group and were reduced by the histone H3 antibody. These results indicated that histone H3 antibody may play a protective role in ALF by inhibiting ferroptosis.

NF‐κB activity is tightly adjusted at multiple levels by positive and negative regulatory factors.^31^ NOD2 is a cytosolic NLR family member that can regulate the NF‐κB signalling pathway through two distinct pathways.^32^ HDAC6 is a member of the class II histone deacetylases and possesses anti‐inflammatory effects.^33^ Li found that HDAC6 inhibition blocked the activation of the NF‐κB signalling pathway by suppressing IĸB phosphorylation in LPS‐induced acute lung injury.^34^ Many studies have proven that extracellular histones can activate the NF‐κB signalling pathway in macrophages.^35^

We further investigated whether there was a connection among NOD2, HDAC6, the NF‐κB signalling pathway and ferroptosis. The NOD2 agonist MDP and HDAC6‐siRNA were applied to histone H3‐treated RAW264.7 cells. The results showed that histone H3 upregulated the level of ferroptosis in RAW264.7 cells. Furthermore, when stimulated with MDP in the H3 group, NOD2, HDAC6, p‐P65, IκBα, Fe^2+^ and ROS levels were significantly increased and the expression of GSH and GPX4 was markedly decreased. When treatment with HDAC6‐siRNA in H3 group, HDAC6, p‐P65, IκBα, Fe^2+^ and ROS levels were reduced and the expression of GSH and GPX4 was elevated. However, NOD2 level between the HDAC6‐siRNA group and the H3 group was not significantly different. The results showed that NOD2 and HDAC6 may act as upstream signalling molecules that can regulate the NF‐κB signalling pathway and ferroptosis. In addition, our data showed that NOD2 could positively regulate HDAC6 but HDAC6 may have a weak impact on NOD2.

**FIGURE 7:**
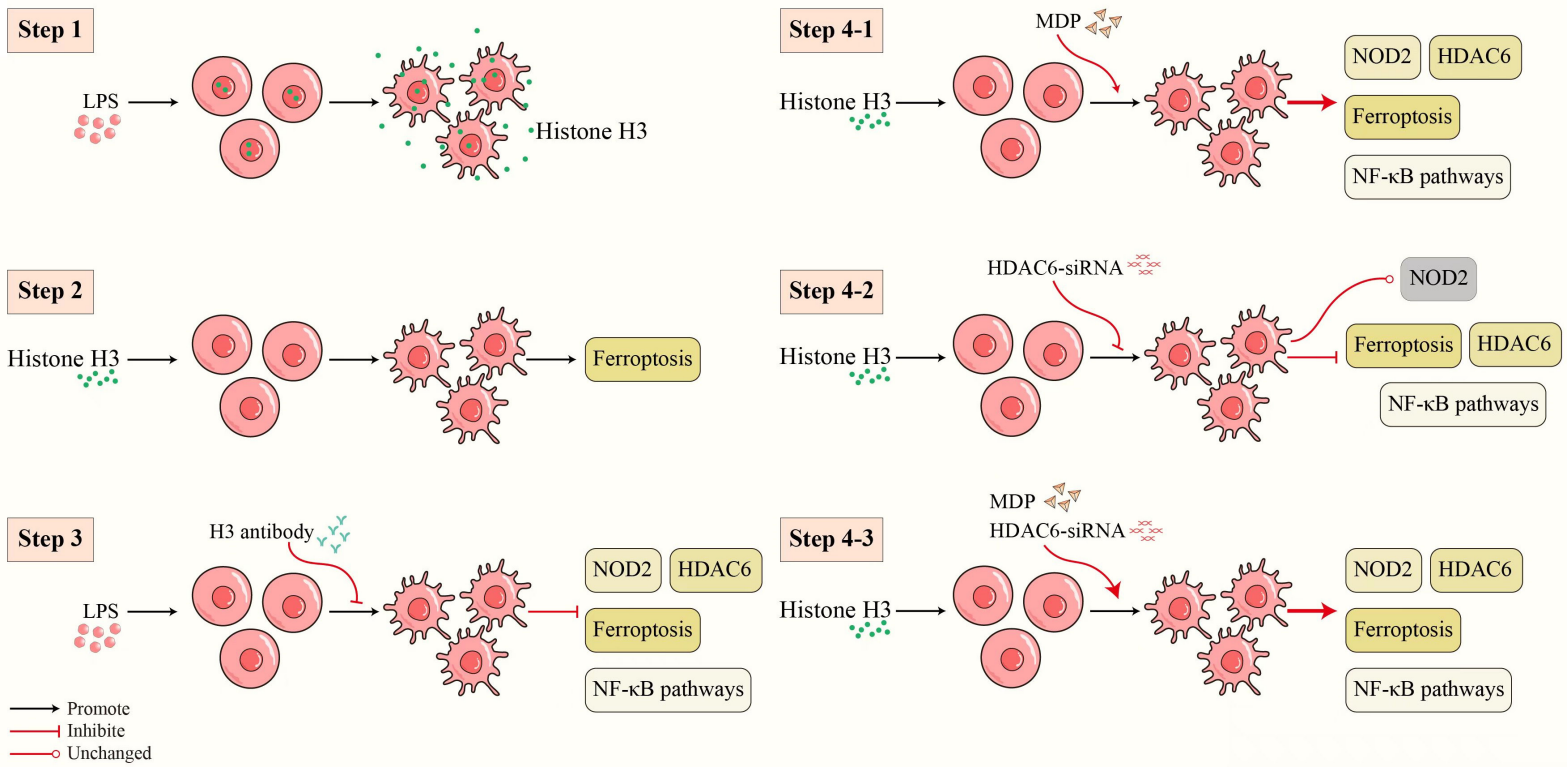
Graphical abstract of the RAW264.7 cells experiment. (Step 1) Histone H3 was released into the extracellular environment when LPS was stimulated. (Step 2) Histone H3‐induced ferroptosis. (Step 3) Histone H3 antibody inhibited NOD2, and HDAC6 levels, the NF‐κB pathway and ferroptosis in LPS‐stimulated cells. (Step 4–1) The NOD2 agonist MDP aggravated t NOD2, and HDAC6 levels, the NF‐κB pathway and ferroptosis in histone H3‐treated cells. (Step 4–2) HDAC6‐siRNA reduced the expression of HDAC6, the NF‐κB pathway and ferroptosis, but had no effect on NOD2 in histone H3‐treated cells. (Step 4–3) MDP increased NOD2, HDAC6 levels, the NF‐κB pathway and ferroptosis in RAW264.7 cells administered histone H3 and HDAC6‐siRNA.

Briefly, the basic process and main findings of cell experiment were summarized and shown in Figure 7.

In summary, extracellular histone H3 induced by LPS could cause ferroptosis in ALF. Administration of histone H3 antibody rescued the mice from the severe liver injury caused by D‐Gal/LPS and the blockade of ferroptosis activation. Moreover, inhibiting the expression of NOD2 and HDAC6 may reduce the levels of the NF‐κB signalling pathway components and ferroptosis in ALF. Further investigation is needed to identify accurate targets of ferroptosis, which will contribute to novel treatment strategies for acute liver failure.

## AUTHOR CONTRIBUTIONS

**Qian Chen:** Conceptualization (equal); formal analysis (equal); methodology (equal); validation (equal); writing – original draft (equal). **Qingqi Zhang:** Conceptualization (equal); formal analysis (equal); methodology (equal); validation (equal); writing – original draft (equal). **Pan Cao:** Formal analysis (equal); methodology (equal); validation (equal). **Chunxia Shi:** Methodology (equal); validation (equal). **Luyi Zhang:** Formal analysis (equal); methodology (equal); validation (equal). **Luwen Wang:** Conceptualization (equal); methodology (equal). **Zuojiong Gong:** Conceptualization (equal); project administration (equal); supervision (equal); visualization (equal); writing – review and editing (equal).

## CONFLICT OF INTEREST

The authors declare that they have no conflicts of interest.

## Data Availability

All data are contained within this manuscript. The data that support the findings of this study are openly available in [repository name, eg ‘figshare’] at reference number.
